# *Ex Situ* Culturing Experiments Revealed Psychrophilic Hydrogentrophic Methanogenesis Being the Potential Dominant Methane-Producing Pathway in Subglacial Sediment in Larsemann Hills, Antarctic

**DOI:** 10.3389/fmicb.2018.00237

**Published:** 2018-02-21

**Authors:** Hongmei Ma, Wenkai Yan, Xiang Xiao, Guitao Shi, Yuansheng Li, Bo Sun, Yinke Dou, Yu Zhang

**Affiliations:** ^1^SOA Key Laboratory for Polar Science, Polar Research Institute of China, Shanghai, China; ^2^School of Life Sciences and Biotechnology, Shanghai Jiao Tong University, Shanghai, China; ^3^College of Electrical and Power Engineering, Taiyuan University of Technology, Taiyuan, China; ^4^State Key Laboratory of Ocean Engineering, Shanghai Jiao Tong University, Shanghai, China; ^5^Institute of Oceanography, Shanghai Jiao Tong University, Shanghai, China

**Keywords:** hydrogenotrophic methanogenesis, East Antarctic, subglacial ecosystem, *mcrA* gene, *ex situ* cultivation, climate change

## Abstract

It was recognized only recently that subglacial ecosystems support considerable methanogenic activity, thus significantly contributing the global methane production. However, only limited knowledge is available on the physiological characteristics of this kind of methanogenic community because of the technical constraints associated with sampling and cultivation under corresponding environmental conditions. To elucidate methanogenesis beneath the glacial margin in East Antarctic Ice Sheet, we took an integrated approach that included cultivation of microbes associated with the sediment samples in the lab and analysis of *mcrA* gene therein. After 7 months of incubation, the highest rate of methanogenesis [398 (pmol/day)/gram] was observed at 1°C on a supply of H_2_. The rates of methanogenesis were lower on acetate or unamended substrate than on H_2_. The rates on these two substrates increased when the temperature was raised. Methanomicrobiales predominated before and after prolonged incubation, regardless whether H_2_, acetate, or unamended substrate were the energy source. Therefore, it was inferred that psychrophilic hydrogenotrophic methanogenesis was the primary methane-producing pathway in the subglacial ecosystem we sampled. These findings highlight the effects of temperature and substrate on potential methanogenesis in the subglacial sediment of this area, and may help us for a better estimation on the Antarctica methane production in a changing climate.

## Introduction

Although the Antarctic subglacial ecosystem has been commonly considered barren and persistently cold (Wadham et al., 2008), it influences the global climate (Cavicchioli, 2015; Gulick et al., 2017). Beneath the Antarctic Ice Sheet, organic carbon is estimated at 21000 Pg (Wadham et al., 2012) and could be degraded by several microbial processes with and without oxygen involved, especially in the wet-based ice sheet basal beds (Bell, 2008; Christner et al., 2008; Hodson et al., 2008). The organic carbon beneath the ice sheet may have a marine, glaci-marine, and crustal sedimentary source (Wadham et al., 2012). Aerobic respiration and reaction with sulfide minerals deplete the dissolved oxygen in the shallow sediment (Wadham et al., 2008), supporting the anaerobic metabolism of organic carbon in deep sediment beneath the ice sheet. In subglacial sediment, methanogenesis is regarded as the last step of carbon metabolism and has recently attracted significant attention (Wadham et al., 2008, 2012; Boyd et al., 2010; Stibal et al., 2012b; Telling et al., 2015; Michaud et al., 2017) because of the high impact of methane as a greenhouse gas (Conrad et al., 2002). The inferred methane hydrate reservoir beneath the Antarctic Ice Sheet is comparable to that in the Arctic region and could constitute a previously neglected component of the global methane hydrate inventory with a potential to act as a positive feedback on climate warming during ice-sheet wastage (Koven et al., 2011; Wadham et al., 2012).

The methanogenic communities and their activities in the subglacial ecosystem, especially in the ice-sheet margin, are influenced by climate change in terms of temperature. Depending on the type of cells, methanogens generate energy by producing methane from H_2_+CO_2_ and/or simple organic compounds, e.g., formate, acetate, methanol, ethanol (Ferry, 2010). Temperature can control decomposition processes in permafrost, thus controlling the abundance and composition of the substrates used by methanogens (Treat et al., 2014). The Q_10_-value of microbial respiration in soil, which represents the microbial sensitivity to temperature changes, was negatively correlated to the temperature changes, especially in cold environments (below 25°C) (Hamdi et al., 2013). A study of Arctic peat soil showed a shift from the hydrogenotrophic Methanobacteriales to Methanomicrobiales and a shift from the acetoclastic Methanosarcinaceae to Methanosaetaceae along with a bacterial community shift corresponding to temperature gradient (Tveit et al., 2014). During the thawing process of a permafrost soil site, both methane emission and oxidation were increased, and the methanogenic community switched from hydrogenotrophic into partly acetoclastic (McCalley et al., 2014). Thus, the methanogenic community reflects the local climate conditions, which requires our attention to perform long-term observation. However, since the *in situ* observation is technically challenging, an *ex situ* examination on the methanogenic community along with multiple temperature and substrate settings would be a desirable alternative.

In the past years, methanogens were widely discovered in the sub-ice sheet environments in polar areas, such as the Greenland ice sheet, Robertson Glacier, Lower Wright Glacier, Russell Glacier, and John Evans Glacier (Boyd et al., 2010; Stibal et al., 2012a,b; Dieser et al., 2014; Telling et al., 2015; Michaud et al., 2017). However, the long-term effect of temperature to the methanogenic activity and community, especially in the East Antarctic subglacial area, remains unknown. A long-term *in situ* observation would always provide the most accurate information, but this approach is constrained by various technical difficulties. Alternatively, an *ex situ* approach, which we applied, allowed us to monitor the methanogenic activity and community dynamics driven by temperature and nutrient shifts over a period of 200 days. For subglacial sediment sampling, we chose a glacial margin in East Antarctica, where the transition zone extends from the subglacial to proglacial environment and is particularly sensitive to the climate-forcing environmental changes. This study was intended to help us understand the potential mechanisms of methane production and to what extent the temperature changes influence methanogenesis in the subglacial sediments from East Antarctica.

## Materials and Methods

### Study Site and Sample Collection

The study site (76°16′11.85′′E, 69°24′57.93′′S) was located on the Ingrid Christensen Coast of Princess Elizabeth Land, East Antarctica, near to the Prydz Basin. At this site, the mean daytime air temperature in winter ranges between -15 and -18°C, and in summer (December, January, and February), it is approximately 0°C. In addition, the temperature can exceed by 4°C and even by 10°C sometimes (Hodgson et al., 2001). Samples were collected in the Polar Plateau ice sheet margin near Huaxi peninsula in Larsemann Hills by the 28th Chinese National Antarctic Research Expedition in January 2012, when the mean air temperature was 2°C. A meltwater stream drained from the front of the ice sheet, and a subglacial sediment was collected from fissures in the ice-sheet margin, where the sediment was covered by 1-m deep snow-ice layers (Supplementary Figure S1) and stored in a sterile sealed bag (CLEANWRAP, South Korea) with air removed. The sediment sample was transported frozen to the lab and maintained at -20°C until further processing. The snowmelt from supraglacial channel system was collected in a 250-ml sterile polypropylene bottle and maintained at 4°C during and after transport to the lab.

### Chemical Analysis

The sediment’s pH was measured using the slurry technique (Herbold et al., 2014) and pH meter (FE20, Mettler Toledo, Columbus, OH, United States). The moisture content was analyzed with a gravimetric soil water method after drying 20 g of sediment at 105°C (Barrett et al., 2004). Five grams of the sample was thawed and centrifuged at 10000 × *g* to obtain the pore water (Ankley and Schubauer-Berigan, 1994) for conducting ion analysis through ion chromatography (MIC, Metrohm, Herisau, Switzerland). The ions in the snowmelt water were similarly analyzed. Fifty grams of the sample was dried for 3 days in a vacuum freeze dryer (Alpha 2-4/LSC-16, Martin Christ, Osterode am Harz, Germany) and subjected to a microstructural analysis with an X-ray diffractometer (Beijing University Micro Structure Analytical Laboratory, Beijing, China) and element quantification with a wavelength dispersive X-ray fluorescence spectrometer (Beijing University Micro Structure Analytical Laboratory, Beijing, China). The inorganic carbon content was analyzed using a Dionex ion chromatography system (ICS 3000, Thermo Scientific, Waltham, MA, United States). The total carbon and total organic carbon (TOC) were analyzed using a TOC-VCPN system (TOC-VCPN, Shimadzu, Japan).

### DNA Extraction and *mcrA* Gene Clone Library Construction

In this study, the DNA was extracted from the sediment sample using a sodium dodecyl sulfate (SDS)-based method following the previously described protocol (Zhou et al., 1996), and the crude nucleic acids were then purified using the Cycle Pure Kit (Omega Bio-Tek, Norcross, GA, United States) (Li et al., 2012). Methyl-coenzyme M reductase (Mcr) catalyzes the reductive demethylation of methyl-coenzyme M (CH_3_-S-CoM) to methane (CH_4_) with the electrons donated by coenzyme B (HS-CoB) (Ferry, 2010). The *mcr* gene is exclusive to the methanogens, except for the methane-oxidizing archaea, and shows mostly congruent phylogeny to the 16S rRNA gene (Steinberg and Regan, 2009). Thus, *mcrA* (methyl-coenzyme M reductase α-subunit) gene analysis is commonly used to examine methanogen communities (Yang et al., 2014). A partial sequence of *mcrA* gene was amplified with the primer pair mlas/mcrA-rev (Steinberg and Regan, 2008). The PCR amplification was performed using the following reaction mix: 30–100 ng DNA, 20 nM of each primer, 5 μl of 10X Ex Taq Buffer (Takara, Japan), 200 μM dNTP (Takara, Japan), 1.25 U ExTaq polymerase (Takara, Japan), 2.5 g/L Bovine Serum Albumin (New England Biolabs, Ipswich, MA, United States), and water to give a final volume of 50 μl. Amplification was performed in a thermal cycler (model 2720, Applied Biosciences, United States) with the following PCR cycling program: initial denaturation at 95°C for 3 min, 30 cycles comprising denaturation at 94°C for 1 min, annealing at 55°C for 1 min, and extension at 72°C for 1 min, and a final extension at 72°C for 10 min. The PCR product was purified using a Gel Extract Kit (Omega Bio-Tek, Norcross, GA, United States). The DNA fragments were then ligated into pMD 18-T vector plasmids (Takara, Japan), transformed into DH5α competent cells (TransGen Biotech, Beijing, China) and grown overnight at 37°C (Li et al., 2012). Subsequently, PCR was used to test the presence of the inserted DNA fragment in the randomly selected clones. Clones with inserted DNA of the appropriate size were sequenced with an ABI 3730 × 1 DNA Analyzer (Sangon, Shanghai, China).

### Methanogen Phylogenetic Analysis

In the *mcrA* gene clone library established from the raw subglacial sediment, 83 clones were analyzed and the obtained sequences were grouped into operational taxonomic units using mothur^1^ with a cut-off value of 84% for the species (Yang et al., 2014). The sequences from this study were aligned with the reference sequences using ClustalW in MEGA 6.0. A phylogenetic tree was constructed using FastTree (Version 2.1.3, ML Model: Jones-Taylor-Thorton) (Price et al., 2010) and visualized with MEGA 6.0 (Tamura et al., 2013).

### *Ex Situ* Culturing Experiments

The incubation experiment was performed using the Hungate technique (Miller and Wolin, 1974) in a Gloveless Anaerobic Chamber (Coy Laboratory Products, Grass Lake, MI, United States). Two types of carbon sources were supplied separately: acetate (sodium bicarbonate, sodium acetate; labeled group A) and CO_2_+H_2_ (sodium bicarbonate, H_2_; labeled group H). A control culture (labeled group C), which did not include an extra carbon source besides sodium bicarbonate, was performed under the same condition. In brief, 4 ml of the pre-cultured slurry [1:1 (v/v) of the sediment and basal salt medium without substrate mixed and cultured at 1°C overnight to homogenize the slurry] was added to 11 ml of the basal salt medium (Zhang et al., 2010) (modified for NaCl, 10 g/L; Na_2_SO_4_, 0 g/L; 5 ml of the NaHCO_3_ solution), including a trace element mixture, NaHCO_3_ solution, vitamin mixture, thiamin solution, and vitamin B_12_ solution (Widdel and Bak, 1998) in a 38 ml glass serum bottle (Wheaton glass serum bottle; Sigma–Aldrich, St. Louis, MO, United States), which created a headspace of 23 ml. For group A, 10 mM sodium acetate (working concentration) was added to the bottle. Subsequently, 25 μl of 10% Na_2_S was added to the bottle after the headspace of the bottle was flushed with N_2_ to create a reduced condition for groups A, C, and H. For group H, a gas-tight gas bag (Leiqi, Shanghai, China) with 0.5 L of H_2_ was connected to the bottle for H_2_ supply. Every condition was run in triplicate, and the bottles were incubated statically at 1, 4, and 12°C and covered with aluminum foil to maintain dark conditions.

The methane in the headspace was sampled with a gas-tight syringe (100 μl, Hamilton, Reno, NV, United States) and analyzed with gas chromatography (GC-2010 Plus, Shimadzu, Japan) after shaking the bottle for 30 s. The GC was equipped with an RTx-5 column (30 m × 0.25 μm × 0.25 mm) at an oven temperature of 50°C. A flame ionization detector at 250°C was used with N_2_ (99.999%) as the carrier gas. Methane was quantified according to the standard sample prepared with methane (99.999%). The headspace pressure of the bottle was detected when sampled for gas analysis. A 0.3-ml slurry sample was collected with a syringe and analyzed for pH with a pH test paper (range from 5.5 to 9.0), and DNA was extracted from 0.3-ml slurry with the SDS-based method described above. The concentration of methane dissolved in the slurry was calculated according to Henry’s law with 1.4 mM methane solubility in the medium with 1% salinity and 1 bar partial pressure of methane (Yamamoto et al., 1976). The total methane per bottle was calculated in mol units according to the ideal gas law, and the methane production rate was calculated according to the increased methane normalized for sediment mass and time increases (time from 107–236 days for groups A and C and 94–222 days for group H).

The H_2_ in group H was sampled with a gas-tight syringe (100 μL, Hamilton, Reno, NV, United States) and analyzed by gas chromatography (GC-14B, Shimadzu, Japan). The GC was equipped with a TDX-02 column at an oven temperature of 100°C and a thermal conductivity detector at 120°C. Argon gas (99.999%) was used as the carrier gas. H_2_ was quantified according to the standard sample.

The acetate in group A was analyzed under UV light (210 nm) using high-performance liquid chromatography (HPLC; Agilent, 1200 series) installed with column 100-5-C18 (Kromasil, Sigma–Aldrich). The sample was filtered using a 0.45-μm filter before analysis.

### *McrA* Gene Quantification

A quantitative PCR (q-PCR) analysis was performed to quantify the *mcrA* copies before incubation and at a later stage of incubation (224 days for groups A and C and 222 days for group H) based on a previously described method (Steinberg and Regan, 2009). Briefly, an *mcrA* gene fragment from the subglacial sediment was amplified with the primer mlas/mcrA-rev (Steinberg and Regan, 2008) and cloned into pMD 18-T vector plasmids (Takara, Japan). The *mcrA* fragment was sequenced (GenBank: KR871852). The plasmid with the cloned *mcrA* gene was 3163 bp. It was transformed into DH5α competent cells (TransGen Biotech, Beijing, China). The plasmid concentration was quantified using a spectrophotometer (NanoDrop 2000, Thermo Scientific, Waltham, MA, United States). The copy number of the *mcrA* gene was calculated according to the concentration (C) of plasmid as follows: copy number/μl DNA = 6.02 × 10^23^ × C (g × μl^-1^) × 660^-1^ × 3163^-1^ bp. The plasmid DNA was 10-fold diluted in ultrapure water (Sangon, Shanghai, China) to create a dilution series from 8.867 × 10^8^ to 8.867 × 10^2^ copies per μl.

A SYBR Green fluorophore was employed for the q-PCR, using 10 μl of SYBR Green Prim Mix Taq II (2X) (Takara, Japan), 0.4 μl of Rox Reference Dye II (50X) (Takara, Japan), 1 μl of mals/mcrA-rev primer each (10 μM), 6.6 μl ultrapure water (Sangon, Shanghai, China), and 1 μl DNA. The samples and standards were amplified on the same plate in triplicate. The q-PCR was run on a Fast Real-Time PCR System (ABI 7500, Applied Biosciences, United States) under the following PCR conditions: 95°C for 30 s, 40 cycles of denaturation at 95°C for 5 s, annealing at 55°C for 30 s, extension at 72°C for 1 min, and image capture during annealing. A melt curve analysis was performed to ensure the specific amplification under these conditions: 95°C for 15 s, 60°C for 1 min and 0.5°C increments every 10 s up to 95°C, with images captured during the increase. The results were accepted only at *R*^2^ > 0.99, 90 < eff% < 110.

## Results

### Study Site and Sample Characteristics

The subglacial sediment from the fissure was dark brown (Supplementary Figure S1) and composed primarily of clay. It contained large amounts of Si (29.5%) and Fe (4.24%) and small amounts of P (0.124%) and Ca (1.37%) (Supplementary Table S1), reflecting the siliceous nature of the study site. The moisture content was as high as 38.46%, which was consistent with melt-water flowing in front of the ice sheet during sample collection. The sediment was slightly acidic (pH 6.51), and the TOC content was as high as 2.0%. The sulfate and nitrate concentrations in the pore water of the sediment were 1.019 and 1.215 mM, respectively, and in the snowmelt were 0.010 and 0.004 mM, respectively (**Table 1** and Supplementary Table S1). The study site was located at the margin of the ice sheet, which was regarded as the extension of the sediment beneath the ice sheet, which remains covered by snow-ice for a long winter time.

**Table 1:** Geochemical characteristics of the samples in this study.

Sample	pH	Moisture	TOC	Na^+^	Mg^2+^	Ca^2+^	Cl^-^	SO_4_^2-^	NO_3_^-^
		%	%	mM	mM	mM	mM	mM	mM
Subglacial sediment	ND	38.46	2.0	ND	ND	ND	ND	ND	ND
Porewater	6.51	ND	ND	3.110	2.492	7.081	2.312	1.019	1.215
Snowmelt	5.82	ND	ND	0.174	0.017	0.004	0.216	0.010	0.004

*TOC, total organic carbon; ND, not detected*.

### *Ex Situ* Methanogenic Activities

**FIGURE 1:**
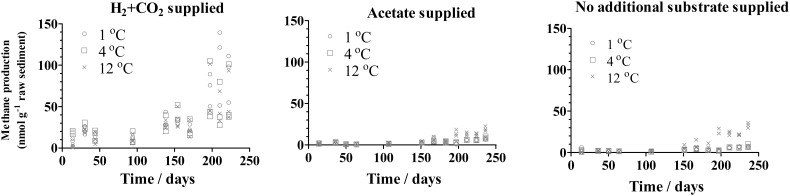
Methane production in subglacial sediment during the incubation in triplicate with different substrates at 1°C (o), 4°C (□), and 12°C (x).

Methane production was detected shortly after H_2_ was supplied to the inoculum at all temperatures applied in this study (1, 4, and 12°C), whereas a delayed methane production was detected after 3 months with supplied acetate or in the presence of unaugmented substrate (**Figure 1**). For the group supplied with H_2_, the initially produced methane appeared to be consumed during the approximately first 30–50 days. Increased methane was produced when H_2_+CO_2_ was supplied (37.3 ∼ 110.1 nmol/g raw sediment at 222 days) compared to the production when acetate was supplied or in the presence of unaugmented substrate (6.4 ∼ 22.7 nmol/g raw sediment and 6.3∼ 36.1 nmol/g raw sediment, respectively, at 236 days). Moreover, H_2_ was consumed under all conditions in group H, whereas acetate was not consumed in group A (Supplementary Figure S2).

**FIGURE 2:**
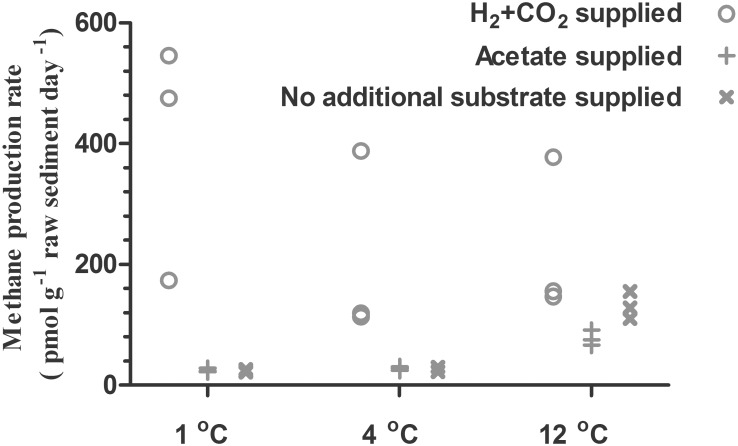
Methane production rates in subglacial sediment incubated in triplicate at different temperatures with different substrates.

The H_2_-supported methanogenic activity appeared to be the highest at an incubation temperature of 1°C (mean value of the triplicate: 398 (pmol/g)/d) and decreased to 207 and 227 (pmol/g)/d as the incubation temperature increased to 4 and 12°C, respectively (**Figure 2**). In contrast, the acetate-supported and substrate-unamended methanogenic activity showed a positive correlation with temperature. The rates of acetate-supported methanogenic activity were 23, 25, and 131 (pmol/g)/d at 1, 4, and 12°C, respectively (**Figure 2**), and the rates of substrate-unamended methanogenic activity were 25, 29, and 78 (pmol/g)/d at 1, 4, and 12°C, respectively (**Figure 2**). Supplied H_2_ stimulated methane production whereas supplied acetate did not have any noticeable effect on it (**Figures 1**, **2**).

Other environmental factors were also monitored during the incubation period. With supplied H_2_+CO_2_, the pH levels decreased at the beginning of incubation (Supplementary Figure S3), whereas with supplied acetate and unaugmented substrate, the pH levels decreased initially and then increased during methane production (Supplementary Figure S3).

### Methanogen Diversity

**FIGURE 3:**
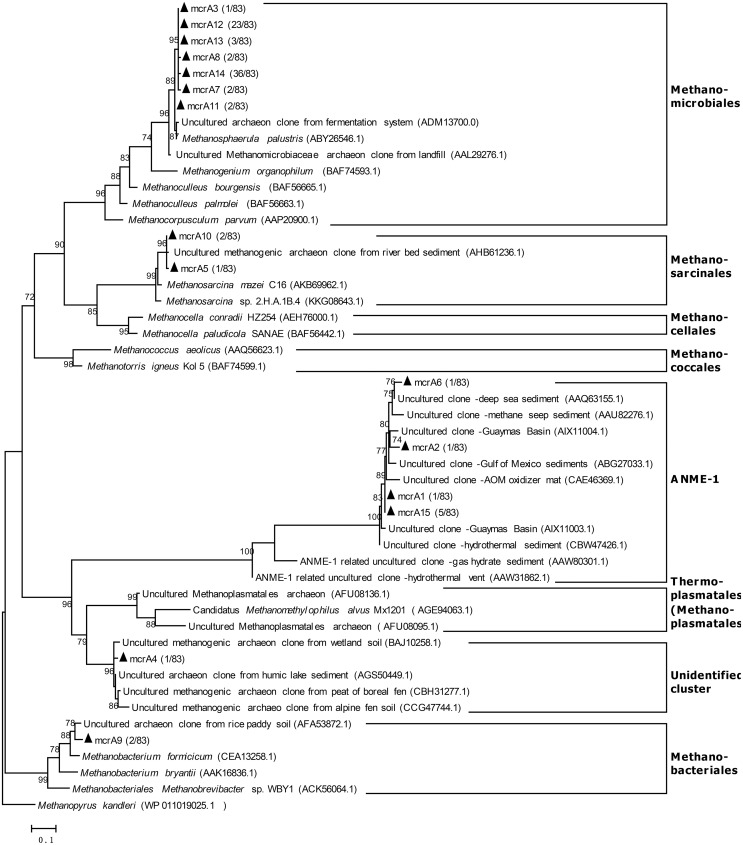
Phylogenetic tree based on translated partial amino acid sequences of the *mcrA* gene from raw Antarctic subglacial sediment. The sequences from this study and reference sequences were aligned with ClustalW in MEGA 6.0. The tree was constructed with FastTree (Version 2.1.3, ML Model: Jones–Taylor–Thorton) and visualized with MEGA 6.0. The scale bar represents a difference of 0.1 substitutions per site. The *mcrA* gene sequences marked with black triangles were derived from this study. The number in parentheses indicates the clone proportion.

Eighty-three clones were analyzed in the *mcrA* clone library (coverage was 92.8%) from the raw subglacial sediment, and 69 clones (83.1%) were classified as Methanomicrobiales (**Figure 3**). According to the National Center for Biotechnology Information (NCBI), the closest sequence was from *Methanosphaerula palustris* E1-9C (ABY26546.1), which shared 99% identity based on its amino acid sequence. This species was isolated from a minerotrophic fen peatland; it can use H_2_+CO_2_ and formate to produce methane, but cannot use acetate for methane production (Cadillo-Quiroz et al., 2009). Three clones (3.6%) were classified as Methanosarcinales, which share 99% identity with sequences from a river bed sediment (AHB61236.1) (Buriankova et al., 2013). Two clones (2.4%) were related to the sequences from a rich paddy soil (AFA53872.1) (Daebeler et al., 2013) and were grouped into Methanobacteriales. One clone (1.2%) was related to the sequences from a boreal fen peat (CBH31277) (Yrjälä et al., 2011), wetland soil (BAJ10258.1) (Narihiro et al., 2011), humic lake sediment (AGS50449.1) (Youngblut et al., 2014), and alpine fen soil (CCG47744.1) (Franchini and Zeyer, 2012), which were grouped into an unidentified cluster. Eight clones (9.6%) were related to the archaeal anaerobic methane oxidizers group 1 (ANME-1) sequences from a deep-sea sediment (AAQ63155.1) (Hallam et al., 2003), with 97% shared identity, and an anaerobic methane oxidizer mat (CAE46369.1) (Kruger et al., 2003) from the Guaymas Basin (AIX11003.1) (Lever and Teske, 2014).

### Methanogen Growth during Incubation

**FIGURE 4:**
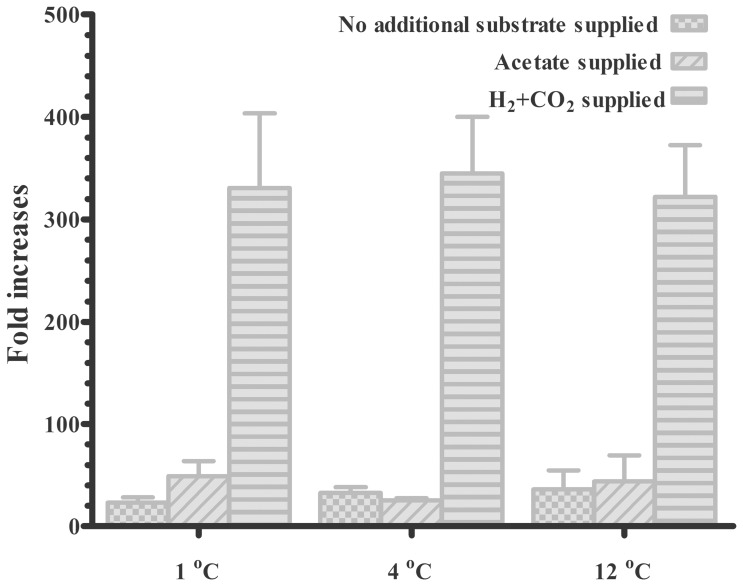
Increase in *mcrA* gene copy number in subglacial sediment after incubation for 7 months. The *mcrA* gene copy number in the original sediment was 2.3 × 10^4^ copy/g sediment according to the q-PCR results. (mean ± SEM, triplicate).

The *mcrA* copy number in the subglacial sediment was estimated for approximately 7 months before and after culture (**Figure 4**). The *mcrA* gene copy number in the original subglacial sediment was 2.3 × 10^4^ copies/g. After an incubation period of 7 months, the *mcrA* gene copy number increased in all groups and showed a greater increase with supplied H_2_ + CO_2_ than with supplied acetate and unamended substrate. With supplied H_2_ + CO_2_, a greater than 300-fold increase was observed at all temperatures. The difference between methane production at different temperatures was small, with a slightly reduced amount at 12°C (322-fold) compared with that at 1°C (330-fold) and 4°C (345-fold). With unamended substrate, the increases were positively correlated with incubation temperatures (23-, 33-, and 36-fold increases at 1, 4, and 12 °C, respectively). The supplied acetate promoted greater increases at 1°C than at 4 and 12°C with 49-, 25-, and 44-fold increase in methane production, respectively. However, because of the technical bias within Q-PCR analysis, as shown as the error bars in **Figure 4**, the difference between incubations with same substrate but at different temperatures was not significant.

## Discussion

### Hydrogenotrophic Methanogenesis as a Dominant Methane-Producing Pathway

Based on the activity tests and community analysis, hydrogenotrophic methanogenesis was inferred as a dominant methane-producing pathway in the subglacial sediment analyzed in this study. Firstly, a hydrogenotrophic methanogenic population was already well developed in the original sediment as demonstrated by the initial methane production rate on supplied H_2_ and CO_2_ (**Figure 1**). Secondly, the growth of methanogens was highly stimulated in the H_2_-supplied group compared with the acetate-supplied and control groups, regardless of the incubation temperature (**Figure 4**). Furthermore, the results from the *mcrA* gene clone library confirmed that the dominant group of methanogens before and after incubation belonged to the order of Methanomicrobiales, closely related to *Methanosphaerula palustris* (**Figure 3**), which utilizes H_2_ and CO_2_ to produce methane (Garcia et al., 2006; Cadillo-Quiroz et al., 2009). This is in accordance with the previous findings. From the 16s rRNA genes analysis, methanogens closely related to *Methanosphaerula palustris* were detected in both Lower Wright Glacier and Russell Glacier sediment (Stibal et al., 2012b). Even after acetate was supplied for 7 months, Methanomicrobiales were still dominant in the methanogenic community (Supplementary Figure S4), suggesting that methane was mainly produced through hydrogenotrophic methanogenesis even when incubating with acetate or without treatment. We made a similar observation with certain *in situ* observations. For example, data from the basal ice of Greenland Ice Core Project (GRIP) (Souchez et al., 2006) and Greenland Ice Sheet Project 2 (GISP2) (Miteva et al., 2009) demonstrated isotopically lighter δ^13^C-CH_4_ values characteristic of methane produced from H_2_ and CO_2_ (Whiticar, 1999). Both the isotopic and genomic signatures revealed that in Subglacial Lake Whillans in West Antarctica, the sub-ice-sheet methane was produced through the biological reduction of CO_2_ using H_2_ as electron donor (Michaud et al., 2017). A likely explanation for this is that the standard Gibbs’ free energy obtained from the hydrogenotrophic methanogenesis is one order of magnitude higher than that obtained from the acetotrophic methanogenesis, especially at low temperature (Supplementary Figure S5). The predominance of Methanomicrobiales has been reported in other glacial ecosystems as well, such as Robertson Glacier and Russell Glacier (Hamilton et al., 2013; Telling et al., 2015), which supports the idea that the hydrogenotrophic methanogenesis is globally well adapted to cold temperature. Thus, as long as the H_2_ bioavailability is sufficient in subglacial environments, the H_2_-fueled methanogenesis is the major source of methane. Moreover, the shift between H_2_-supporting into acetate-supporting methanogenic communities could not be achieved in short period even if the substrate changes, thus the *ex situ* experiments could reflect the original nature of the methanogenesis *in situ*. For example, in Stibal’s experiments, the methanogenesis in Russell Glacier sediment was elevated after H_2_ and CO_2_ were added compared to that acetate was added, while the methanogenesis in Lower Wright Glacier and John Evans Glacier behaved differently (Stibal et al., 2012b). And in Boyd’s experiment, the methanogens from Robertson Glacier were clustered into Methanosarcinales and the methanogenic activities were restored after acetate was added (Boyd et al., 2010). Although not examined in this study, an assessment of the bioavailability of H_2_ and other energy sources for methanogenesis in the subglacial ecosystem would be important.

In subglacial sediment, H_2_ could be derived from biotic or abiotic processes. Fermenting bacteria can degrade organic matter into low-molecular weight products, such as ethanol, butyrate, succinate, lactate, propionate, formate, acetate, and hydrogen. The pH normally drops in this process. In addition, certain hydrogen-producing bacteria can consume light-weight molecules and produce H_2_ (Schink, 1997; Whiticar, 1999; Conrad, 2002) for use by methanogens. This is similar to the process that occurs in hydrogen-facilitated ecosystems (Schink, 1997; Nealson et al., 2005). It was previously speculated that H_2_ is likely produced by radiolysis or serpentinization in the McMurdo Dry Valleys of East Antarctica and was detected in the brine of an ice-sealed Antarctic lake (Murray et al., 2012). A recent study demonstrated the H_2_ production at 0°C probably through the reaction of water with mineral surface silica radicals formed during rock comminution. And this H_2_ production could be sufficient to support the previously measured rates of methanogenesis under a Greenland glacier (Telling et al., 2015). Besides, although the bacterial community was not investigated in our study, it was estimated that 2% of the TOC in the sediment could lead to a considerable amount of bacterial hydrogen production. Meanwhile, because of the low sulfate concentration (1 mM), sulfate reducers could compete only weakly with methanogens, thus accounting for a dominance of hydrogenotrophic methanogens. In the present study, in the absence of added acetate or other substrate, methane production accompanied by an increase in pH was delayed for 3 months (Supplementary Figure S3). Thus, methanogenesis when either acetate or no substrate was supplied involved two steps: organic carbon degradation to H_2_ accompanied by a drop in pH, followed by methane production accompanied by an increase in pH.

### Temperature Effect on Methane Production

The hydrogenotrophic methanogenic community showed high activity at low temperature. When the incubation temperature was increased from 1 to 4 or 12°C, no increase but a slight through highly variable reduction in the rate of methane production was observed (**Figure 2**). This phenomenon may be partly explained by a linear decrease in the bioavailable energy (Supplementary Figure S5), and may also indicate that the methanogens involved were psychrophiles. According to the *in situ* record, the mean monthly air temperature at Huaxi peninsula in Larsemann Hills, which was close to our sampling site, was approximately 0°C in summer (December, January, and February) and -15°C ∼-18°C in winter (Hodgson et al., 2001). In a study of Russell Glacier sediment, a decrease in the rate of methanogenesis in the presence of H_2_ was observed when the incubation temperature was raised to 10°C (Stibal et al., 2012b).

On the other hand, an increase in temperature showed a positive effect on the acetate-supported methanogenic activities, especially at 12°C (**Figure 2**). Similar results were observed in the sediments from Robertson Glacier, where acetate-metabolizing methanogenesis was the primary methane-producing pathway, the methane productivity at 15°C was a few times higher than that at 4°C (Boyd et al., 2010). This could be partly because the energy generated from the acetotrophic methanogenesis was positively correlated to temperature (Supplementary Figure S5). These observations strongly suggest that the hydrogen-metabolizing and acetate-metabolizing methanogens may have different responses to the temperature changes, thus different evolutionary paths after living in a cold environment for a long time. Moreover, considering that the hydrogenotrophic methanogenesis was the dominant process even when acetate was supplied, the hydrogen availability was critical to the methane production rate. In the incubations without added H_2_, H_2_ could have been derived from the degradation of organic carbon (**Table 1**) (Schink, 1997; Whiticar, 1999; Conrad, 2002). This process is documented to be positively affected by temperature when the substrate is sufficient (Westermann, 1999). This could be another reason for positive correlation of the calculated net production rate of methane with temperature (**Figure 2**). Organic carbon under the Antarctic ice sheet and near the Ingrid Christensen Coast of Princess Elizabeth Land, Prydz Bay, was presumably derived from the shallow marine, continental, or lagoon areas (Ivanov, 1989). In general, the endogenous methane production from organic carbon in the subglacial sediment was positively correlated with temperature, which suggests a potential feedback effect on global warming, since methane is a powerful greenhouse gas.

### Evaluating the Potential for Methane Source Beneath Glacial Ecosystems

**FIGURE 5:**
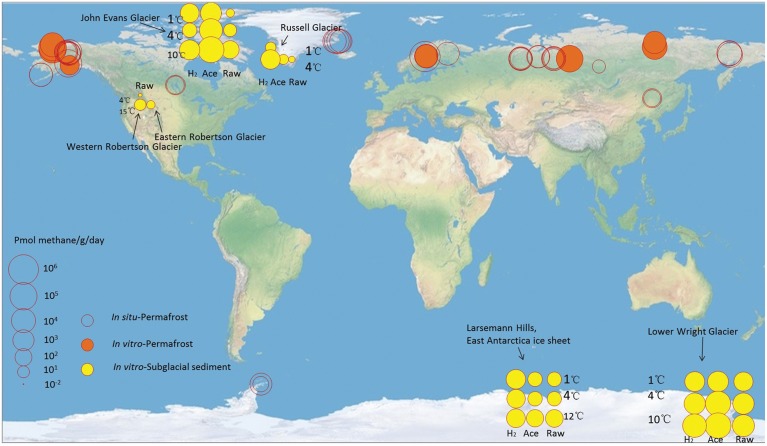
Methane production rate ((pmol/g)/day) in subglacial sediment and permafrost. For the subglacial sediment, the *ex situ* incubation temperature and the substrate (H_2_: H_2_ supplied; Ace: acetate supplied; Raw: no additional substrate supplied) concentrations were obtained from corresponding experiments. For the permafrost sediment, the *ex situ* data were obtained from corresponding experiments. The *in situ* data were obtained from the *in situ* static chambers. The *in situ* flux values were converted from (mg/m^2^)/day to (pmol/g)/day according to the depth of active layer and 2 g/cm^3^ density of soils. Data were derived from this study and references (Supplementary Table S2).

Although substantial subglacial methane reservoirs have been predicted (Wadham et al., 2012), information describing the release of subglacial methane and its influence on atmospheric methane concentrations remains scarce (Siegert et al., 2012; Fricker et al., 2013; Talalay, 2013). To our knowledge, all available data are based on *ex situ* incubation. This study is the first report on methane production in the ice sheet margin of East Antarctica. At this sampling site, the substrate-unamended methanogenic activity was 10^2^ ∼ 10^3^ (pmol/g)/day, which is in the middle range compared to that of the other glaciers (**Figure 5** and Supplementary Table S2). This number is insignificant compared with methane production in other types of ecosystems, such as rice fields (10^5^ ∼ 10^6^ (pmol/g)/d) (Yuan et al., 2012) and permafrost (10^3^ ∼ 10^6^ (pmol/g)/d for *ex situ* study and 10^1^ ∼ 10^5^ (pmol/g)/d for *in situ* study) (**Figure 5**). This may be explained by the cold temperature, and partly by the lack of organic carbon input through photosynthetic processes in subglacial environments, especially in the multi-year ice environments (Boetius et al., 2015).

The ice sheet margin provides a window to explore such methane sources. A greater understanding of such source will contribute to improved estimations on the methane budget in subglacial ecosystems. If we take the estimated volume of the subglacial basal zone as 20 km^3^ (Boetius et al., 2015), the annual methane production in the subglacial ecosystem would be estimated to be in the order of 10^5^ ∼ 10^6^ kg, which is 5 ∼ 6 orders of magnitude lower than that in the wetlands, the largest methane source on Earth (Reeburgh, 2007). However, in the subglacial environment, especially in the shallow sediment layer, oxygen from the release of air bubbles in melting basal ice makes possible presence of oxygen, nitrate, Fe(III) and sulfate, which are more favorable to be taken by microorganisms as electron acceptors compared to carbon. Hydrogen is therefore firstly consumed by the nitrate reducers and sulfate reducers before methanogens. Since in most of the *ex situ* experiments, including ours, the batch-style cultivation applied and the oxygen intrusion has been largely avoided, the *in situ* methanogenic activity may be overestimated by just looking at the data observed in lab. Meanwhile, to model the methane budget, the processes to consume methane also need to be addressed. Aerobic methanotrophy can effectively remove > 99% of the methane before it reaching the atmosphere in Subglacial Lake Whillans sediments, west Antarctic (Michaud et al., 2017). Interestingly, ANME-1s, which can consume methane anaerobically (Hinrichs et al., 1999; Lloyd et al., 2006), were also detected in the *mcrA* clone library in the subglacial sediment (**Figure 3**). Although the co-existence of ANMEs and methanogens has been reported previously in other ecosystems (Kendall and Boone, 2006; Lloyd et al., 2006; Lloyd et al., 2011; Lever and Teske, 2014), the AOM processes in subglacial ecosystems have not been reported previously and requires further investigation.

## Conclusion

In this study, the hydrogenotrophic Methanomicrobiales were detected as the key players in the East Antarctic Ice Sheet margin to produce methane at the rate of 10^2^ ∼ 10^3^ (pmol/g)/day, depending on the hydrogen availabilities. The methanogens therein have been well adapted to cold environment, thus the temperature increase did not stimulate the methanogenesis, especially the hydrogenotrophic methanogenesis. Future research should focus on the comprehensive understanding of both bacterial and archaeal communities as well as the effect from other environmental parameters, such as pressure, which could help to further illustrate the methane production process. These findings highlight the effect of chemical and physical conditions on methanogenesis in subglacial sediments.

## Accession Numbers

The *mcrA* gene sequences were deposited in the NCBI GenBank database under the accession numbers KR871810–KR871925.

## Author Contributions

HM and YZ designed the experiments. WY performed the incubation experiment and molecular clone experiment. GS and YD sampled the sediment from the East Antarctica. YL and BS provide field and logistical support and theoretical guidance. WY, YZ, XX, and HM analyzed the data and wrote the manuscript.

## Conflict of Interest Statement

The authors declare that the research was conducted in the absence of any commercial or financial relationships that could be construed as a potential conflict of interest.

## Footnotes

1 http://www.mothur.org/
