# Supplementary material for: Ex Situ Culturing Experiments Revealed Psychrophilic Hydrogentrophic Methanogenesis Being the Potential Dominant Methane-Producing Pathway in Subglacial Sediment in Larsemann Hills, Antarctic
Source: Front Microbiol. 2018 Feb 21;9:237. doi: 10.3389/fmicb.2018.00237 (PMC5826372; doi:10.3389/fmicb.2018.00237)
Supplement: Supplementary file 1 [file Image_1.PDF]

Supplementary Material

Journal: Frontiers in Microbiology

In vitro demonstration on the psychrophilic hydrogentrophic methanogenesis from subglacial sediment in Larsemann Hills, Antarctic

Hongmei Ma[a*], Wenkai Yan[b*], Xiang Xiao[b], Guitao Shi[a], Yuansheng Li[a], Bo Sun[a], Yinke Dou[c], Yu Zhang[d#]

[a] *SOA Key Laboratory for Polar Science, Polar Research Institute of China, Shanghai, China*

[b] *School of Life Sciences and Biotechnology, Shanghai Jiao Tong University, Shanghai, China*

[c] *College of Electrical and Power Engineering, Taiyuan University of Technology, Taiyuan, China*

[d] *State Key Laboratory of Ocean Engineering, Shanghai Jiao Tong University, Shanghai, China*

[*]These authors contributed equally to this work

[#]Corresponding to: Yu Zhang, Email: zhang.yusjtu@sjtu.edu.cn. Tel: +86 (0)21 34207208. Fax: +86 (0)21 34207205.

This file includes:

Table S1-S2

Figure S1-S5

Table S1: Geochemical characteristics of subglacial sediment

	Element and Mineral formula	%, w/w, cool dried sediment
Mineral content in sediment	SiO_2（Quartz）	46
	$(Na,Ca)Al(Si,Al)_3O_8$（Albite）	23
	$KAlSi_3O_8$（Microcline）	10
	$(Mg,Al,Fe)_6(Si,Al)_4O_{10}(OH)_8$（Clinochlore）	2
	$KMg_3(Si_3Al)_{10}(OH)_2$（Phlogopite）	14
	$CaMg(CO_3)_2$（Dolomite）	1
	$Al_2Si_2O_5(OH)_4$（Kaolinite）	2
	$Fe^{+2}Fe^{+3}O_4$（Magnetite）	1
	$Al_{3.2}Ca_{3.4}Fe_{4.02}K_{0.6}Mg_6NaSi_{12.8}O_{44}(OH)_4$（Amphibole）	2
Element in sediment	C	2.74
	N	0.598
	O	48.1
	F	0.180
	Na	0.782
	Mg	1.04
	Al	6.89
	Si	29.5
	P	0.124
	S	0.159
	Cl	0.099
	K	3.45
	Ca	1.37
	Fe	4.24

Table S2. Methane production rate in subglacial sediment and permafrost

Study Methods	Site Location	Latitude	Longitude	Ecosystem classification/ supplied substrate	Average methane flux (mg /m^2/day) for *in situ* measure or methane production rate (pmol/g/day) for *in vitro* measure	Soil temperature or incubation temperature (℃)	Active layer depth(cm)	Reference
In situ measure	Yukon Delta, Alaska, US	60.75	-161.75	Permafrost fen	143.6	9	100	(Olefeldt et al. 2013)
In situ measure	Thompson, Manitoba, Canada	56.52	-98.7	Permafrost fen	5.7	9.1	120	
In situ measure	Thompson, Manitoba, Canada	56.52	-98.7	Permafrost fen	16.3	6.8	120	
In situ measure	Zackenberg, Greenland	74.5	-21	Permafrost fen	54		60	
In situ measure	Zackenberg, Greenland	74.5	-21	Permafrost fen	204		60	
In situ measure	Zackenberg, Greenland	74.5	-21	Permafrost fen	316		60	
In situ measure	Zackenberg, Greenland	74.5	-21	Permafrost fen	103.2	6.5	60	
In situ measure	Kolyma River floodplain, Sakha Republic, Russia	69.6	161.33	Permafrost fen	196	5	47	
In situ measure	Lek Vorkuta, Komi Republic, Russia	67.38	63.37	Permafrost fen	20	4.2	50	

In situ measure	Lek Vorkuta, Komi Republic, Russia	67.38	63.37	Permafrost fen	59	4.3	49	
In situ measure	Lek Vorkuta, Komi Republic, Russia	67.38	63.37	Permafrost fen	75.2	5.1	54	
In situ measure	Lek Vorkuta, Komi Republic, Russia	67.38	63.37	Permafrost fen	80.2	5.5	56	
In situ measure	Lek Vorkuta, Komi Republic, Russia	67.38	63.37	Permafrost fen	136	7.1	60	
In situ measure	Yamal Peninsula, Tyumen Oblast, Russia	68.13	71.7	Permafrost fen	59.5		80	
In situ measure	Yamal Peninsula, Tyumen Oblast,Russia	68.13	71.7	Permafrost fen	195.4		150	
In situ measure	Kolyma River floodplain, Sakha	69.6	161.33	Permafrost fen	320		47	
In situ measure	Arctic Coastal Plain, Alaska, U	70	-150	Permafrost fen	11.4		45	
In situ measure	Arctic Coastal Plain, Alaska, U	70	-150	Permafrost fen	44.3		45	
In situ measure	Arctic Coastal Plain, Alaska, U	70	-150	Permafrost fen	78.8		45	
In situ measure	Tanana River floodplain, Alaska	64.63	-148.33	Permafrost fen	22		200	
In situ measure	Chersky, Sakha Republic, Russi	68.83	161.67	Permafrost fen	281.1		55	
In situ measure	Taz, Tyumen Oblast, Russia	67.35	78.91	Permafrost fen	18.7	9.1	30	

In situ measure	Taz, Tyumen Oblast, Russia	67.35	78.91	Permafrost fen	26.5	9.2	150	
In situ measure	Taz, Tyumen Oblast, Russia	67.35	78.91	Permafrost fen	47.4	9	20	
In situ measure	Taz, Tyumen Oblast, Russia	67.35	78.91	Permafrost fen	76.4	9.3	35	
In situ measure	Taz, Tyumen Oblast, Russia	67.35	78.91	Permafrost fen	96.3	7	33	
In situ measure	North Slope, Alaska, US	70.16	-148.37	Permafrost fen	190		41	
In situ measure	North Slope, Alaska, US	69.69	-148.75	Permafrost fen	75		42	
In situ measure	Brooks Range, Alaska, US	69.29	-148.73	Permafrost fen	78		45	
In situ measure	North Slope, Alaska, US	69.52	-148.55	Permafrost fen	34		53	
In situ measure	Stordalen, Sweden	68.37	19.05	Permafrost fen	57.6	5.9	50	
In situ measure	Stordalen, Sweden	68.37	19.05	Permafrost fen	158.4	6.2	76	
In situ measure	Yukon Delta, Alaska, US	60.75	-161.75	Permafrost fen	143.6		100	
In situ measure	FM,Kandu River,Great Xing'an Mountains,China	51.13	125.14	montane permafrost	8.64		90	(Liu et al. 2015)
In situ measure	NFM,Kandu River,Great Xing'an Mountains,China	51.13	125.14	montane permafrost	2.64		90	

In situ measure	CP,Bøttemyra wetland in Finnmark (northern Norway)	69.41	29.12	discontinuous permafrost	63		40	(Liebner et al. 2015)
In situ measure	MM1,Ardley Island， King George Island	-62.13	-58.56	tundra marsh	0.3216		20	(Zhu et al. 2014)
In situ measure	WM1,Ardley Island， King George Island	-62.13	-58.56	tundra marsh	11.376		20	
In situ measure	Tura， Siberia,Russia	64.16	100.13	continuous permafrost	0.0992		20	(Morishita et al. 2014)
In vitro measure	Lena Delta, Siberia	72.22	126.28	Permafrost	20000	-3		(Wagner et al. 2007)
In vitro measure					3000	-6		
In vitro measure	Alaska	65.4	-149.04	Permafrost	100000	5		(Waldrop et al. 2010)
In vitro measure	Stordalen mire	68.21	18.49	Permafrost	1600000	14		(Lupascu et al. 2012)
In vitro measure	Igarka Siberia	67.3	86.36	Permafrost	75000	4		(Metje and Frenzel 2007)
In vitro measure	Barrow, Alaska	71.17	-156.37	Permafrost	100000	8		(Chowdhury et al. 2015)
In vitro measure					100000	4		
In vitro measure					10000	-2		

In vitro measure	Larsemann Hills,East Antarctica	-69.25	76.16	subglacial sediment	23	1		This study
In vitro measure					24	4		
In vitro measure					131	12		
In vitro measure				Acetate supplied-subglacial sediment	24	1		
In vitro measure					28	4		
In vitro measure					107	12		
In vitro measure				H_2 supplied subglacial sediment	398	1		
In vitro measure					207	4		
In vitro measure					227	12		
In vitro measure	Lower Wright Glacier, Antarctica	-77.25	163	subglacial sediment	310	1		(Stibal et al. 2012)
In vitro measure					1200	4		
In vitro measure					2000 *	10		
In vitro measure				Acetate supplied-subglacial	700 *	1		

In vitro measure				sediment	19000 *	4		
In vitro measure					30000 *	10		
In vitro measure				H_2 supplied subglacial sediment	500 *	1		
In vitro measure					1200 *	4		
In vitro measure					6000 *	10		
In vitro measure	Russell Glacier, Greenland	67.03	-50.1	subglacial sediment	0.18	1		
In vitro measure					1.9	4		
In vitro measure				Acetate supplied-subglacial sediment	0.7 *	1		
In vitro measure					4.7 *	4		
In vitro measure				H_2 supplied subglacial sediment	5.7 *	1		
In vitro measure					379.1 *	4		
In vitro measure	John Evans Glacier, Canadian Arctic	79.38	-74.23	subglacial sediment	2.9	1		
In vitro measure					15	4		

In vitro measure					158 *	10		
In vitro measure				Acetate supplied-subglacial sediment	1154 *	1		
In vitro measure					4700 *	4		
In vitro measure					39000 *	10		
In vitro measure				H_2 supplied subglacial sediment	290 *	1		
In vitro measure					30 *	4		
In vitro measure					500 *	10		
In vitro measure	Eastern Robertson Glacier, Canada	50.44	-115.2	subglacial sediment	0.216	4		(Boyd et al. 2010)
In vitro measure				subglacial sediment	3.024	15		
In vitro measure	Western Robertson Glacier, Canada			subglacial sediment	1.224	4		
In vitro measure				subglacial sediment	7.656	15		

*: Data from the figures in the reference paper.

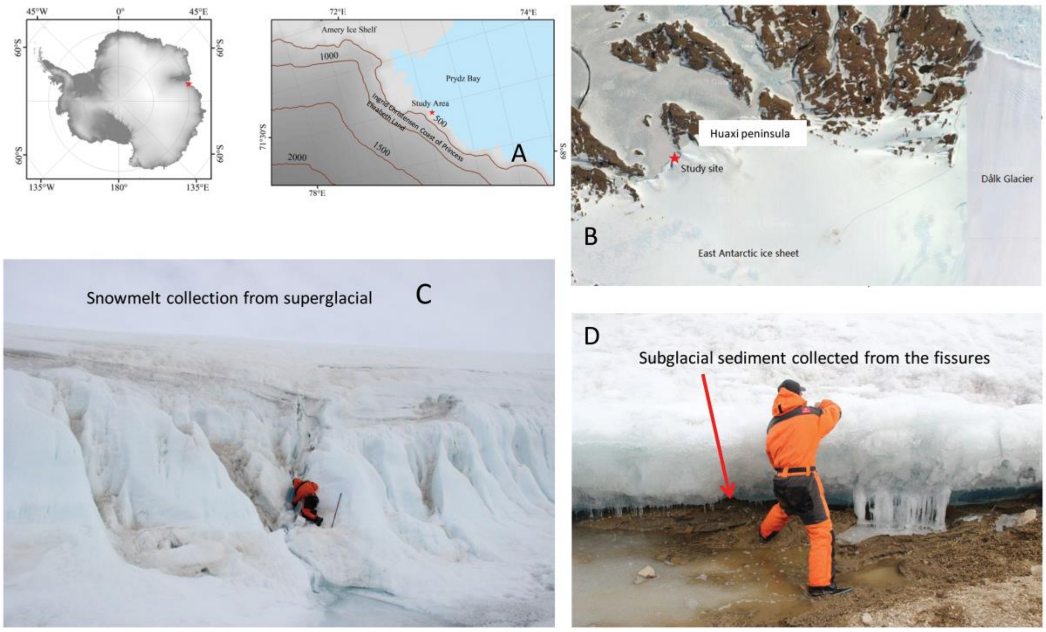

Figure S1: Maps showing the location of sampling site in East Antarctica ice sheet. A. The Ingrid Christensen Coast of Princess Elizabeth Land, East Antarctic. B. The study site near to the Huaxi Bandao, in the ice sheet margin (modified from Google Earth). C. Snowmelt collection from superglacier. D. Subglacial sediment collection from the fissures.

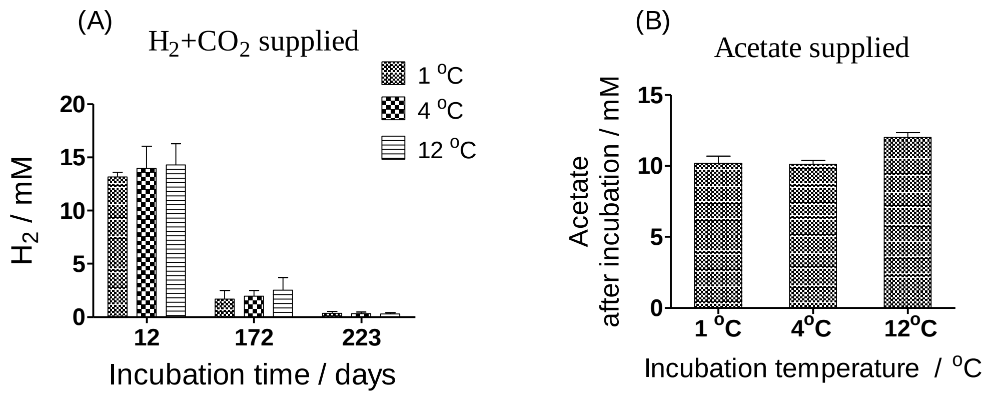

Figure S2: H_2 and acetate detection for the incubation. (A) H_2 amount in the gas bag after H_2 was supplied for 12 days, 172 days and 223 days (mean±SEM, n=3). (B) Acetate amount after incubated for 222 days in group with acetate supplied (mean±SEM, n=3).

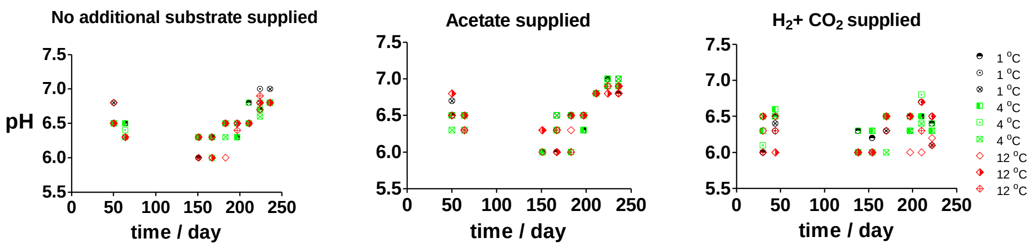

Figure S3: The pH detection during incubation at different temperatures with different substrates.

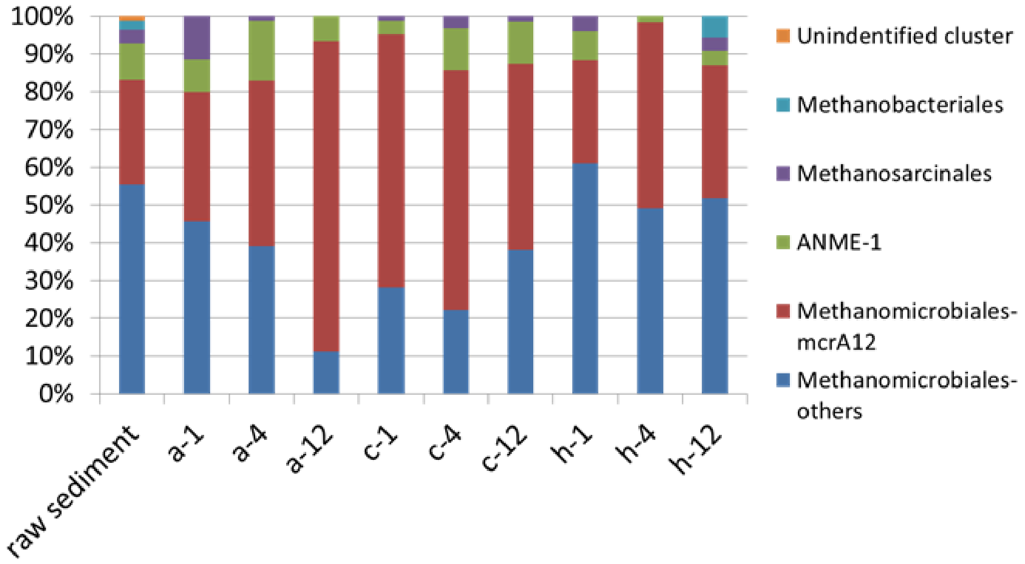

Figure S4: Diversity of the *mcrA* gene before and after incubation. The *mcrA* gene clone library was constructed with the primer pair mcra-mlas/rev. Raw sediment represents the raw subglacial sediment. a-1, a-4, and a-12 represent the cultures with the highest methane production rates at 1, 4, and 12 ℃ under supplied acetate; c-1, c-4, and c-12 represent the cultures with the highest methane production rates at 1, 4, and 12 ℃ under unaugmented substrate; and h-1, h-4, and h-12 represent the cultures with the highest methane production rates at 1, 4, and 12 ℃ under supplied H_2. The *mcrA* gene-mcrA12 in the raw sediment was also dominant after incubation.

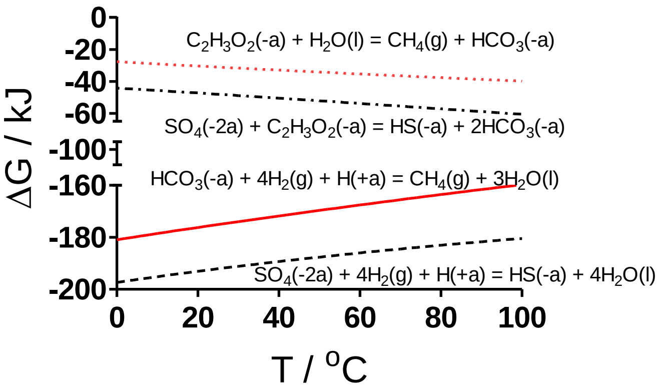

Figure S5: Delta G of related reaction in subglacial sediment. This was calculated using the HSC chemistry 5 software.

References:

Boyd ES, Skidmore M, Mitchell AC, Bakermans C, Peters JW (2010) Methanogenesis in subglacial sediments Environ Microbiol Rep 2:685-692

Chowdhury TR et al. (2015) Stoichiometry and temperature sensitivity of methanogenesis and CO_2 production from saturated polygonal tundra in Barrow, Alaska Global Change Biol 21:722-737 doi:10.1111/gcb.12762

Liebner S, Ganzert L, Kiss A, Yang SZ, Wagner D, Svenning MM (2015) Shifts in methanogenic community composition and methane fluxes along the degradation of discontinuous permafrost Front Microbiol 6:356 doi:10.3389/fmicb.2015.00356

Liu X, Guo YD, Hu HQ, Sun CK, Zhao XK, Wei CL (2015) Dynamics and controls of CO_2 and CH_4 emissions in the wetland of a montane permafrost region, northeast China Atmos Environ 122:454-462 doi:10.1016/j.atmosenv.2015.10.007

Lupascu M, Wadham JL, Hornibrook ERC, Pancost RD (2012) Temperature sensitivity of methane production in the permafrost active layer at Stordalen, Sweden: a comparison with non-permafrost northern wetlands Arct Antarct Alp Res 44:469-482 doi:Doi 10.1657/1938-4246-44.4.469

Metje M, Frenzel P (2007) Methanogenesis and methanogenic pathways in a peat from subarctic permafrost Environ Microbiol 9:954-964 doi:10.1111/j.1462-2920.2006.01217.x

Morishita T, Matsuura Y, Kajimoto T, Osawa A, Zyryanova OA, Prokushkin AS (2014) CH_4 and N_2O dynamics of a Larix gmelinii forest in a continuous permafrost region of central Siberia during the growing season Polar Science 8:156-165 doi:10.1016/j.polar.2014.01.004

Olefeldt D, Turetsky MR, Crill PM, McGuire AD (2013) Environmental and physical controls on northern terrestrial methane emissions across permafrost zones Global Change Biol 19:589-603 doi:10.1111/gcb.12071

Stibal M et al. (2012) Methanogenic potential of Arctic and Antarctic subglacial environments with contrasting organic carbon sources Global Change Biol 18:3332-3345 doi:DOI 10.1111/j.1365-2486.2012.02763.x

Wagner D, Gattinger A, Embacher A, Pfeiffer E-M, Schloter M, Lipski A (2007) Methanogenic activity and biomass in Holocene permafrost deposits of the Lena Delta, Siberian Arctic and its implication for the global methane budget Global Change Biol 13:1089-1099 doi:10.1111/j.1365-2486.2007.01331.x

Waldrop MP, Wickland KP, White Iii R, Berhe AA, Harden JW, Romanovsky VE (2010) Molecular investigations into a globally important carbon pool: permafrost-protected carbon in Alaskan soils Global Change Biol 16:2543-2554 doi:10.1111/j.1365-2486.2009.02141.x

Zhu RB, Ma DW, Xu H (2014) Summertime N_2O, CH_4 and CO_2 exchanges from a tundra marsh and an upland tundra in maritime Antarctica Atmos Environ 83:269-281 doi:10.1016/j.atmosenv.2013.11.017
